# Retraction: Development of Alendronate-conjugated Poly (lactic-co-glycolic acid)-Dextran Nanoparticles for Active Targeting of Cisplatin in Osteosarcoma

**DOI:** 10.1038/srep31938

**Published:** 2016-09-16

**Authors:** Ping Liu, Liang Sun, Dong-sheng Zhou, Peng Zhang, Yong-hui Wang, Dong Li, Qing-hu Li, Rong-jie Feng

Scientific Reports 5: Article number: 17387; 10.1038/srep17387 published online: 12 01 2015; updated: 09 16 2016.

The authors of this Article have requested that it be retracted. During attempts to scale up the experiments for clinical studies, we could not reproduce the apoptosis (Figure 4) and cell cycle distribution results reported in the paper. We carried out a detailed intra-departmental investigation and found that the equivalent concentrations of cisplatin (CDDP) in the nanoparticles (PLD) had been miscalculated, and that the MG63 cells were cross-contaminated, leading to significant errors in the experimental results.

All the authors recognize that these errors have affected the validity of the Article and have agreed to retraction. The authors apologize for their carelessness.

